# Community-Based Monitoring in the New Normal: A Strategy for Tackling the COVID-19 Pandemic in Malaysia

**DOI:** 10.3390/ijerph18136712

**Published:** 2021-06-22

**Authors:** Nur Khairlida Muhamad Khair, Khai Ern Lee, Mazlin Mokhtar

**Affiliations:** 1 Institute for Environment and Development (LESTARI), Universiti Kebangsaan Malaysia (UKM), Bangi 43600, Selangor, Malaysia; n.khair@yahoo.com (N.K.M.K.); mazlin@ukm.edu.my (M.M.); 2 Centre for Research and Instrumentation Management (CRIM), Universiti Kebangsaan Malaysia (UKM), Bangi 43600, Selangor, Malaysia; 3 Jeffrey Sachs Center on Sustainable Development, Sunway University, Petaling Jaya 47500, Selangor, Malaysia

**Keywords:** COVID-19, pandemic, mitigation, community-based monitoring, sustainability

## Abstract

In 2020, the COVID-19 pandemic severely impacted the global public health system and led to many deaths worldwide. COVID-19 is highly contagious and can be spread by symptomatic or asymptomatic individuals. As such, determining the risk of infection within a community is difficult. To mitigate the risk of the spread of COVID-19, the government of Malaysia implemented seven phases of the movement control order (MCO) from 18 March to 31 December 2020. However, the socioeconomic cost was substantial despite the effectiveness of the MCO in bringing down cases of infection. As noted by the Prime Minister of Malaysia, the final criterion that should be met is community empowerment. In other words, community-based mitigation measures through which communities unite to contain the pandemic are essential before the completion of the vaccination program. As a measure for controlling the pandemic, mitigation strategies in the new normal should be feasible, practical, and acceptable to communities. In this paper, we present a deliberation of a set of community-based monitoring criteria to ensure health and well-being in communities, such as efficacy, technicality, feedback, and sustainability. The proposed criteria will be instrumental in developing community-based monitoring initiatives to achieve the desired goals in coping with the pandemic as well as in empowering communities to be part of the governance process.

## 1. Introduction

Since the outbreak of the COVID-19 pandemic, the struggle to find effective measures to contain the proliferated infection continues in countries around the world. As the pandemic is unprecedented, no specific playbook exists for determining best practices to end the outbreak. Answers to questions on how to end the outbreak are dependent on the measures implemented by global governments. Thus, countries are taking immediate action to control crowd movement through physical restrictions, such as quarantine, curfew, or lockdown and social and physical distancing. Such measures delay the rampant spread of the outbreak, relieve the pressure on health care systems, and reduce the number of patients until effective medication or vaccine become widely available to everyone [1,2].

Movement restriction has proved effective in reducing the number of cases of infection. In China, massive lockdowns and travel restrictions have reduced the number of positive cases abruptly [2]. Despite this effectiveness, the burden of the pandemic, widely measured in terms of disability-adjusted life years [3,4,5,6], had a significant inevitable consequence on the global economy with heavy movement restrictions on the primary, secondary, and tertiary sectors. As a result, many people have lost their jobs due to the inability of companies to cope with deficits [7]. Moreover, the extreme economic condition has triggered increases in other public health concerns related to mental health, such as anxiety and depression [8]. The ripple effect of the pandemic has presented challenges to governments in structuring policies that form responses to the needs of society.

Governments are exerting their best efforts to ensure that actions taken to curb the COVID-19 outbreak will not burden the people. Moreover, the mitigation of this pandemic remains a long journey; the end to this pandemic has yet to be achieved. In the long run, governments can no longer afford to keep countries in lockdown mode because the economic and social sectors are crucial to the survival of the country. Reducing movement restriction may not be favorable from the health perspective; however, neglecting socioeconomic factors will endanger the economy of a country. Lifting certain restrictions may be the best option for governments to reopen the socioeconomic sectors. In this regard, the cooperation of all parties, especially the people, are vital in flattening the curve. The responsibility for addressing the outbreak lies in the hands not only of governments, authorities, and health sectors, but also of the people.

In this paper, we suggest empowering communities to be part of the governance process in combating the COVID-19 pandemic. Examining Malaysia as a case study, we report how the Malaysian government and people worked together in tackling the COVID-19 pandemic in 2020. This article is structured as follows. In Section 1, we report on the COVID-19 pandemic scenario in Malaysia in 2020 and how the government reacted to contain possible new infections. Section 2 is devoted to discussing the socioeconomic impacts of the pandemic. In Section 3, we provide a new perspective of governance based on evolutionary governance theory and links to the current initiatives by the government. Finally, in Section 4, which is linked to Section 3, we present community-based monitoring as a way forward for the response to the pandemic by incorporating communities into the exit strategy. With that all in place, mitigating the spread of COVID-19 infection among the communities lies with the communities whereby community leaders, through community-based monitoring, can play an important role in collaborating with local authorities to ensure that their areas are COVID-19-free. The deliberated criteria for community-based monitoring will enable close cooperation between community leaders and local authorities in tackling the current global public health crisis.

### 1.1. The COVID-19 Pandemic in Malaysia

The number of positive cases of COVID-19 is increasing on a daily basis and has yet to indicate any sign of recovery. Malaysia, together with other ASEAN countries, endeavors to win the battle as the uncertain fluctuation trends of daily reported cases are worrisome. The Ministry of Health (MOH) of Malaysia analyzes the local transmission of COVID-19 cases using the value of the basic reproductive number (*R*_0_), which refers to susceptibility to a virus at the beginning of an epidemic in the society. On average, a positive case or host of the virus infects several people. For instance, an *R*_0_ value of four indicates that each positive case can spread the disease to four individuals within a certain period. If no attempt is taken to mitigate the spread, the chain of transmission will proceed exponentially, whereas continuous and adequate preventive measures can lessen the value of the effective reproduction number (*R_t_*) over time. Thus, continuously reducing the value of *R_t_* to less than 1 can put an end to an outbreak.

The study recalls the first case reported in Malaysia on 24 January 2020 with only 22 import and close contact cases [9]. Medical teams successfully handled the cases with no record of death, and all patients were discharged from the hospital. However, on 4 March 2020, 14 new cases were reported compared to only three new cases reported on 3 March 2020 [10]. Since then, the number of reported positive cases increased gradually on a daily basis. One of the biggest contributors to the sudden spike of COVID-19 cases was a Brunei citizen who was found positive after attending a religious gathering in Sri Petaling Mosque, Selangor, Malaysia from 27 February to 1 March 2020. The gathering was composed of more than 12,000 participants from Canada, Nigeria, India, Australia, China, and South Korea [11,12]. Within this period, the value of *R_t_* recorded was 3.5. Thus, the government promptly implemented the MCO to prevent further local transmission. The prompt action taken by the government and medical teams successfully reduced the value of *R_t_* to 0.3 as well as the number of new infections and fatalities [13]. The abovementioned scenario constitutes the biggest COVID-19 cluster in Malaysia and the first wave of COVID-19 infection. Unfortunately, 3375 individuals were infected (Malaysian: 2550; foreigners from 28 countries: 825). A total of 34 deaths were recorded, and this cluster officially ended on 8 June 2020.

In mid-April, the number of reported cases gradually decreased to less than 100 cases per day. However, from May to early June, new reported cases pointed to a sudden spike with more than 100 cases of local infections in which non-Malaysians were mostly affected. These cases were attributed to quarantine detainees at immigration detention centers as well as immigrant hotspot areas. From that time until early September, the number of reported cases did not exceed 50 cases per day. However, on 7 September 2020, a sudden spike in new positive cases marked the beginning of the second wave of local infection with a high *R_t_* value of 1.7 followed by increasing cases in the states of Kedah and Sabah. The high *R_t_* value in these two states led to a major impact on the national *R_t_* value [13].

To date, the third wave of the pandemic started on 20 September 2020 with an *R_t_* value of 2.2 [13]. However, the *R_t_* value decreased to 1.5 within four weeks, which indicated that daily cases of COVID-19 were increasing, albeit slowly. As of 31 December 2020, the total number of infected cases was 113,010. A new record of the highest positive cases in one day, 2525, was set; most cases were transmitted locally (Abdullah 2020d), in which Selangor contributed the highest number of cases, followed by Sabah, Johor, and Malacca [14,15].

### 1.2. Movement Control Order

To mitigate the risk of COVID-19, Malaysia implemented the seven phases of the MCO from 18 March to 31 December 2020 (Table 1). Each MCO phase reflected the level of outbreak severity and corresponding mitigation measures recommended by the government. During MCO phases 1 and 2, the government enforced strict mitigation measures as the first step in containing the spread of the virus. This step resulted in the closure of all educational institutions, places of worship, and nonessential sectors. The government permitted operation at full capacity only for the agriculture, manufacturing, and service sectors, which are related to the production of food supplies and essential needs. All restrictions imposed by the government were under the advice of the Malaysian National Security Council (MNSC) and under the Prevention and Control of Infectious Diseases Act 1988 (Act 342) and Police Act 1967 [16].

To contain the spread of the virus, interstate and interdistrict travel was prohibited unless justified. In addition, the head of family was allowed to travel only within a 10 km radius to purchase groceries. To ensure the effectiveness of MCO, the police and military departments collaborated to mount roadblocks at all state and district borders to monitor the movement of the people. Furthermore, Malaysians and noncitizens were prohibited from leaving or entering the country. However, the government permitted Malaysians to return from overseas, but they were subjected to a 14-day quarantine and a host of medical examinations upon arrival. Noncitizens with diplomatic status and permanent residents except for those managing cruise ships were permitted to enter Malaysia. Despite the travel ban, domestic and international flights continued during this period, especially for domestic and international cargo operations.

The MCO was essential in slowing down the outbreak. López and Rodó [17] suggested that approximately 60 days of lockdown can prevent the pandemic from spreading as well as a potential second wave of COVID-19 cases. However, after implementing four weeks of MCO during phases 1 and 2, business owners began to face difficulties in maintaining businesses if operations were further shut down. On 10 April 2020, the government extended the MCO for another two weeks until 28 April 2020. To lessen the impacts of the economic downturn on the people, the government permitted a few economic sectors to operate with strict conditions, such as half capacity. However, social activities within the periods of MCO phases 1 to 3 were prohibited. In phases 4 to 7, the government permitted the resumption of all socioeconomic activities at 100% capacity given compliance to standard operating procedure (SOPs).

**Table 1:** The phases of the movement control order (MCO) in Malaysia.

	1st MCO	2nd MCO	3rd MCO	4th CMCO	5th CMCO	6th RMCO	7th RMCO
Period	18 Mar–31 Mar 2020	1 Apr–14 Apr 2020	15 Apr–28 Apr 2020	29 Apr–12 May 2020	13 May–9 June 2020	10 June–31 Aug 2020	1 Sept–31 Dec 2020
**Economy Activities**							
Agricultural	As usual	As usual	As usual	As usual	As usual	As usual	As usual
Mining & Quarrying	Ordered to close	Ordered to close	50% of workforce allowed to operate	100% workforce allowed to operate	Normal operation with SOP	Normal operation with SOP	Normal operation with SOP
Construction							
Manufacturing	Food production only	Food production only					
Services	Allowed to operate: - Food - Water - Energy - Telco & Internet - Security & defense - Solid waste, sewerage, public cleaning management - Healthcare & medical - Banking & finance - Public transports - Logistics - E-commerce	Allowed to operate - Food - Water - Energy - Telco & Internet - Security & defense - Solid waste, sewerage, public cleaning management - Healthcare & medical - Banking & finance - Public transports - Logistics - E-commerce	Allowed to operate - Food - Water - Energy - Telco & Internet - Security & defense - Solid waste, sewerage, public cleaning management - Healthcare & medical - Banking & finance - Public transports - Logistics - E-commerce	All services allowed except cinemas, gymnasium, and salons	All services allowed except cinemas, gymnasium, and salons	All services allowed to operate with SOP	All services allowed to operate with SOP
**Social Activities**							
Education	Ordered to close	Ordered to close	Ordered to close	Ordered to close	Ordered to close	Only higher institutions remain closed	Only higher institutions remain closed
Religious	Ordered to close	Ordered to close	Ordered to close	Ordered to close	Ordered to close	Allowed to open with SOP	Allowed to open with SOP
Travel	Allowed only within the district	Allowed only within the district	Allowed only within the district	Only interdistrict allowed	Only interdistrict allowed	Restriction only to overseas	Restriction only to overseas
Sports	Not allowed	Not allowed	Not allowed	Only nonphysical contact allowed effective 4 May 2020	Only nonphysical contact allowed	Physical contact sports allowed with SOP	Physical contact sports allowed with SOP
Social gathering	Not allowed	Not allowed	Not allowed	Not allowed	Not allowed	Allowed in less than 250 attendees with SOP	Allowed in less than 250 attendees with SOP

MCO—Movement Control Order; CMCO—Conditional Movement Control Order; RMCO—Recovery Movement Control Order; SOP—Standard Operating Procedure. Sources: [18,19].

The fluctuation trends of positive cases in the country posed a problem for the government because the increase of positive cases in several states and districts continued to pose a high risk of transmission. Although MCO phases 1 to 3 flattened the infection curve, it exerted a huge impact on the national economy. Hence, further reinforcing a full lockdown in the country was considered infeasible. To overcome the situation, the government implemented CMCO, enhanced MCO, targeted enhanced MCO, and administrative enhanced MCO in several targeted areas [18] to control the community transmission of COVID-19 (Table 2).

Areas under CMCO underwent less restriction. In other words, people can commute within their districts, and COVID-19 screening was not compulsory for residents. For example, CMCO phases 4 and 5 allowed the majority of economic activities to operate at full capacity. However, the recently increasing number of cases led the government to implement CMCO with a few adjustments in SOP compared to CMCO phases 4 and 5. The residents within the area were required to work from home, except for those in the essential sectors; social gathering was banned, all education institutions were ordered to close, all places of worship were allowed an attendance of only six managerial personnel; and interdistrict and state travel were banned. Reviewing the current trend of COVID-19 cases in Malaysia, estimating the endpoint of the pandemic is difficult. Hence, the MOH appeals to all parties to play their respective roles in combating the pandemic. Although the MOH and other agencies are carrying out their duties, communities are requested to continue providing support by complying with prescribed SOPs.

## 2. Post-COVID-19: The New Normal

### 2.1. Economic Reboot

**Figure 1:**
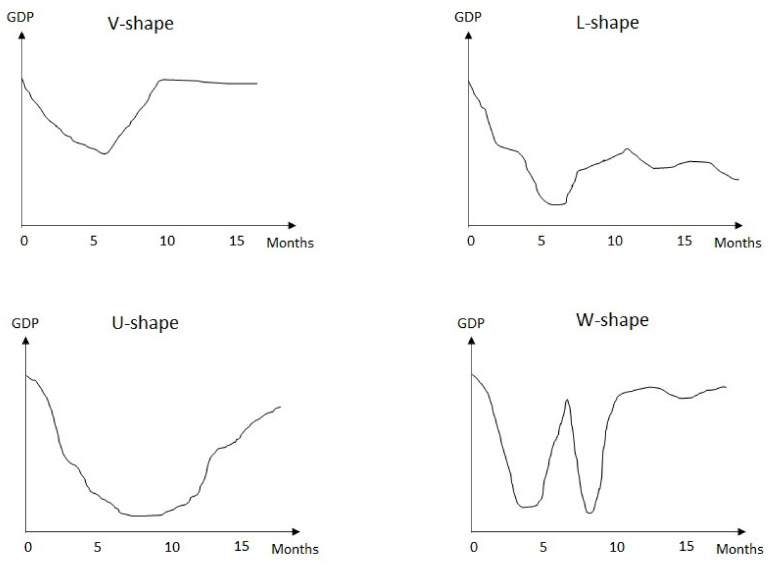
The trends of economic recession and recovery [27].

The economic development at the global level has declined amid the COVID-19 pandemic. Particularly, unemployment rate has increased as a result of social distancing measures, which are intended to suppress the spread of the virus. In addition to the governments’ decision to declare lockdown during the initial wave of the pandemic, businesses are struggling to maintain operations. As a result, many people lost their jobs; unemployment rates continue to increase, service sectors are struggling to survive; and the countries’ economic growth is at stake. Thus, several questions have arisen: “Can the economy recover once the pandemic is over?”, “When will the pandemic end?”, and “What should we expect?” To understand the process of economic reboot postpandemic, the basic trends to grasp are the “LUVW” trends (Figure 1).

The ideal economic reboot is a V-shaped trend that denotes a drastic form of economic recovery and indicates a sharp rebound after the onset of the pandemic. Translating this phenomenon, China [2] and New Zealand experienced a sharp economic downturn during the pandemic [20]. However, both countries have seemingly resumed prepandemic economic activities after the lifting of the social distancing order. Such economy reboot is driven by a substantial shift in economic activities and triggered by a rapid re-adjustment of market demand and business capital expenditure. The rapid recovery in the major aggregate measures of macroeconomics due to the rapid economic change results in a V-shaped recovery. This phenomenon can be considered the best-case scenario after an economy has reached a state of recession.

The second ideal economic reboot features a U-shaped trend, which resembled the V-shaped recovery. In this case, the economy spends a long time in a state of recession rather than rebounds instantly. In this form of recession, a typical cycle ranges from 12 to 24 months to recover. During the pandemic, its impacts on economic growth remain beyond the end of the social distancing order, whereas GDP is recuperating in a gradual manner. Despite the decreased health threats, the economy is far from normal, even if it eventually achieves normalcy. At the beginning of the pandemic, global economic sectors were negatively affected as a whole, including the oil and gas sector. However, the demand for oil and gas significantly dropped because the majority of countries were on lockdown mode, thus leading to a drop in the price of oil in New York at USD 18 per barrel [21]. By May, many countries eased the lockdown restriction and reopened their economy, which resulted in a surge in the demand for oil and other economic sectors [22]. Nevertheless, despite the resumption of economic activities, the pandemic remains a long journey as the total numbers of positive cases and death toll per day continue to increase globally. In other words, recovery from this pandemic may take longer.

The worst-case scenario is the L-shaped trend, which denotes a slow recovery rate, persistent unemployment, and sluggish economic growth. L-shaped recovery occurs after an economic recession marked by a more or less rapid economic downturn without a correspondingly rapid recovery. This type of recovery is the most dangerous form of recession and recuperation [23] because economic growth may exhibit a sharp drop without improvement for a long period. An L-shaped economy reboot will lead to prolonged economic depression. To further translate this phenomenon, countries badly hit by the pandemic due to the collapse of health facilities, high casualties from positive patients, and high unemployment rates due to the struggling economy need to undergo a long recovery state due to the impacts of the pandemic. Sadly, the L-shaped recovery could portray the current macroeconomic performance worldwide.

The fluctuating W-shaped recovery is referred to as a “double-dip recession”, where the economy fluctuates throughout the recovery process. For clarity, a short-lived recovery is observed after economic sectors are reopened followed by another economic downturn with a new wave of COVID-19 infection [24]. The third wave of the pandemic in Malaysia has currently increased the risk of a double-dip recession, despite the announcement of the government of a MYR 250 billion (USD 60.6 billion) economic stimulus package [25]. The objective of the package is to safeguard the welfare of the people and strengthen the economy by supporting individuals and companies facing financial and liquidity constraints [26]. Such a one-off economic injection strategy will push the economy along a path of recovery within a few months to encourage and improve consumer demand (U-shape). However, once the aids are consumed, the scenario could lead to a double-dip recession before the economy can bounce back to a stable state (W-shape).

A recovery in the V-shape trend is observed as a longer shock amplitude or duration. This prediction is reserved for the situation in which the shock is sufficiently mild and unable to impair the country’s financial health. Nevertheless, strong pandemic shocks can result in a permanent unstable state (L-shape) with high unemployment, declining wages and savings, and high rates of financial crises and bankruptcy. Nonetheless, an L-shaped scenario can be avoided if the market demand spikes after a shock. A one-time economic injection strategy will help push the economy along the path of recovery over a period of a few years (U-shape) to encourage and improve consumer demand. With the presented modes of economic recovery, one important question emerges: “What will determine the recovery of the economy?” Importantly, recovery is dependent on the magnitude of the shock and strategies used by governments in introducing policies to mitigate the intensity of the pandemic faced by communities worldwide [27].

### 2.2. Social and Behavioral Changes

The COVID-19 outbreak brought chaos into daily life, thus creating new additional routines or lifestyle that required people to adopt. In addition, it triggered major shifts in the behavior of society, whether temporary or long-term. Amid the pandemic, wearing face masks in public became the new norm. Prevention measures, such as wearing of face masks and hand sanitation, are highly recommended because COVID-19 may present as asymptomatic (i.e., individuals who are infected may not show symptoms). According to the World Health Organization (WHO), people are advised to wear face masks in public to reduce the risk of virus transmission [28]. Furthermore, many countries implemented the compulsory wearing of face masks among citizens, especially in crowded spaces, such as shopping malls, public transport, and religious centers. The Malaysian government imposed compulsory wearing of face masks in public spaces on 1 August 2020. Individuals who fail to comply with the regulation may be sued and pay a compound of at least MYR 1000 (USD 240) under Act 342 [29,30].

Moreover, the landscape of businesses has changed after the implementation of social or physical distancing and avoidance of crowded spaces. The scenario has shifted consumer behavior, such that online shopping has become a choice of convenience [31]. The increased number of online users has been viewed as a good opportunity for retailers to expand their marketing strategies through online platforms instead of conventional stores. However, such a scenario has posed the inevitable impact of the pandemic to the supply chain of businesses. Furthermore, the enforcement of lockdown has disrupted the country’s import and export facilities, which may result in the shortage of product supply and inflate the commodity prices of high-demand products [32]. Other factors that contributed to the shortage of supply during the pandemic were panic buying, which led to hoarding behavior toward essential items, such as food and medical supplies [33]. Experts on behavior have explained that people became panicked when they saw others purchasing items in bulk, which triggered anxiety from being left out [34,35]. Nonetheless, this behavior has been proved temporary, as consumers currently display normal purchase behaviors observed before the implementation of lockdown.

Social or physical distancing not only affects businesses but also causes cultural shifts in adapting to the new norm and in adhering to government mitigation measures. For instance, on 1 July 2020, the Malaysian government established SOPs that limited the number of gathering attendees to only 250 people at a time [36]. Later, on 15 July 2020, the government revised the SOPs and lifted the restriction on the number of attendees. Other SOPs such as wearing face masks, social distancing, temperature screening, and sanitation procedures should be in place [37]. However, such measures should be adjusted to the evolution of the pandemic.

## 3. Calling for Evolutionary Governance

Global governments and public health systems face unprecedented challenges in understanding and governing the unexpected COVID-19 outbreak. In particular, formulating policies, plans, and procedures has proved difficult for governments in anticipation of changes that may emerge. Such changes influence governance mechanisms regarding particular challenges and unprecedented events, which may shift the entire governance framework. Furthermore, limited information and uncertainties of the COVID-19 outbreak have affected governments’ agility and adaptivity in decision making to combat the pandemic. In the present context, agility refers to the speed of the governments’ responses to contain the COVID-19 outbreak, whereas adaptivity entails system-level adjustments in governments, especially in the process of decision making and implementation [38].

With an acknowledgement of the evolving shifts in the new normal governance, this study aims to deliberate on evolutionary governance theory (EGT) as the emerging concept for the mechanisms and communication strategies to be evolved within the governance framework during the pandemic. This theory is an ongoing theorization project by Kristof van Assche et al. [39]. The project aims to understand governance as a coevolution of actor/institution and power/knowledge configurations. Moreover, it is founded on the premise that the whole is a product of evolution in governance, including actors, institutions, organizations, and discourses [39,40]. The authors argued that governance should be recognized as a nested process because they are constantly transforming one another. From the perspective of EGT, unveiling the coordination and configuration between formal and informal institutions is beneficial in elucidating the manner in which institutions are interconnected and transformed [39,40].

**Figure 2:**
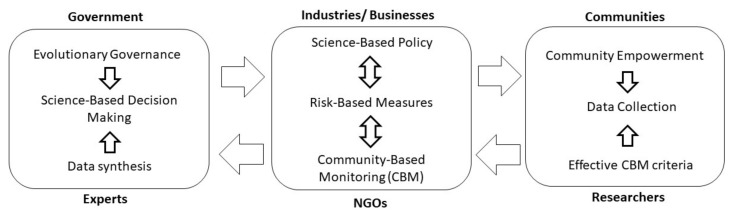
The conceptual approach of evolutionary governance amid COVID-19 pandemic.

The COVID-19 pandemic may prompt further modes of central steering, where such changing modes of steering will subsequently remodel the roles of information, which will then influence power distributions, roles of informality, and actors and balance of participation or representation. In this situation, the government plays a major role in facilitating coordination among various stakeholders. Success in managing a situation or disaster can be achieved using a good governance model that adapts to changes in formal and informal institutions and enables a continuous transformation of power and knowledge configuration between actors [41,42]. Thus, this study incorporates evolutionary governance into a conceptual approach that deals with the volatile, uncertain, complex, and ambiguous nature of COVID-19 pandemic (Figure 2).

### 3.1. Volatility, Uncertainty, Complexity, and Ambiguity

COVID-19 has become a new pandemic worldwide, where many countries face a critical state in managing and controlling the rampant spread of the virus. The pandemic is not only substantially challenging the health care systems and economic sectors but also constantly putting pressure on governments’ actions to formulate sensible and effective strategies in controlling the virus. The fast-changing state can be denoted as “volatility, uncertainty, complexity, and ambiguity” (VUCA), which evokes instant change for sustainability [43].

Volatility refers to unstable and unexpected challenges that emerge within a certain period [44], where changes in the system or impacts of particular changes should be addressed. When the first COVID-19 case was detected in Wuhan in December 2019, the WHO declared the outbreak a pandemic within only a few months (March) [45]. The declaration triggered global responses on the issue as the numbers of positive cases and death rate increased rapidly on a daily basis. Countries then implemented preventive measures, such as quarantine, curfew, and lockdown, to control the unstable situation.

The lack of evidence-based medicine or established articles on managing the pandemic was notable as the virus was novel. Thus, predicting current and future outcomes was difficult. Ongoing clinical research on the development of vaccines is also uncertain regarding the effectiveness, safety, and security of such vaccines. This scenario is referred to as “uncertainty”, where knowledge was unavailable as a result of the hazardous nature of the virus. As basic knowledge about the pandemic was null, the predictability of the spread was low, which reduced the level of confidence in the estimated cause and effect chain [46].

The pandemic is extremely “complex” in nature because it not only affects the health sector but also triggers disastrous socioeconomic and political crises in countries with high rates of infection. Due to the lockdown, the manufacturing, tourism, and transportation industries experienced tremendous losses due to extended lockdown periods [47]. The low production rates of such industries were low, which directly influenced GDP and placed countries at risk of high inflation, unemployment crisis, and decreased hours of work [48,49]. The development of technology and Internet connectivity has promoted the use of smartphones with access to the Internet worldwide. When the pandemic hit, the situation led to social media panic and fear mongering among the public due to misinformation and fake news, panic or mass buying of surgical masks, and bulk purchase of other essential supplies [50,51]. The interconnected impacts of COVID-19 call for collaboration among multiple stakeholders across disciplines to develop effective measures in curbing the impacts of the pandemic on society [52].

Many perspectives can be used to view “ambiguity” in the context of the pandemic. Scientists are hard at work in the race against time to develop vaccines for COVID-19, despite the lack of guarantee about the effectiveness, safety, and security of such vaccines. The unprecedented pandemic offers new challenges as well as opportunities to learn every day, especially in terms of decision making, where policy makers continuously update their countries’ strategies by keeping up with the latest trends. Chen et al. [53] predicted that the virus presents a high probability of mutating significantly into COVID-19 strains with high levels of infections. In virtue of this prediction, the future may be faced with new clusters of COVID-19 that remain undiscovered in the present. Hence, determining the risks of each cluster is vital for governments in formulating effective preventive measures against COVID-19 infections from other strains in the process of reopening the economy.

From the perspective of evolutionary governance, the ongoing volatility information about the current COVID-19 pandemic is a perfect conception of the theoretical source of EGT on the biological theories of evolution, such as autopoiesis (a biological system is a result of the evolution of that system [38,39]. Although the pandemic is uncertain and less predictable, using the EGT may enable transparency in the governance process to explicitly define the complexity and ambiguity of the pandemic.

### 3.2. Science-Driven Policy and Decision Making

In policymaking, governments are focusing on delivering benefits for society at all levels. However, such policies would only be effective if the information available is adequate and accurate. During such an unprecedented and uncertain pandemic, establishing relevant policies is challenging for policymakers due to the lack of time and lack of evidence-based information. The VUCA condition of the pandemic has urged governments to utilize any current information related to the COVID-19 research on decision making. Previous decisions may no longer be the final or best measures. However, the contribution of scientific research in the decision-making process is undeniable.

For instance, the MOH of Malaysia has worked closely with the MNSC in the decision-making process. Daily updates on the ongoing research on the COVID-19 outbreak and current COVID-19 status are key information for decision-makers in strategizing appropriate mitigation measures. In this manner, the decisions of the government are strictly based on facts and factual data [54]. As a result, the Malaysian government implemented MCO and social distancing policy in mid-March [55]. During the MCO, other actors from NGOs and businesses/industries and those apart from government agencies played their roles in supporting the implementation of the policies. For instance, the banking sector provided financial aid to citizens [26], whereas NGOs offered social welfare services to those in need [56]. After the first phase of MCO, the number of reported positive cases slightly decreased, whereas the number of recovered patients increased [57]. The positive trends enabled the economic sectors to resume operations at full capacity in April with strict adherence to the social distancing policy [58]. This approach has proved that using scientific data in decision making, especially during the pandemic, is highly beneficial.

The process of decision making in a science-driven policy requires high-quality and reliable data for analysis and interpretation. During the pandemic, the government is required to act rapidly and make sound decisions within a short period of time. However, conventional methods for acquiring and processing data from different sources are time- and cost-consuming [59]. Hence, the government cannot rely on conventional approaches in handling the pandemic; instead, it can use various efficient big data collection methods, such as smart sensors, mobile applications, wearable devices, and other devices, that have emerged within a short period [60]. Presently, data are collected through screening and monitoring by thermal scanning [61]. Individuals with body temperature exceeding 37.5 °C will be denied entry to premises. Thermal scanning is crucial because it is the first step in constraining potential infected individuals at the point of entry. However, Quilty et al. [62] found that entry or exit screening via thermal scanners is unlikely to be effective due to the duration of the incubation period of the COVID-19 infection.

In addition, other data sources, such as tracking systems, collect data through mobile applications to detect the movement of citizens and for contact tracing. In Malaysia, the federal government launched the MySejahtera application as a monitoring tool to empower the citizens in self-assessing their health risk against COVID-19. In return, the application provides the MOH with relevant information for early detection [62]. Data collected through the MySejahtera application include name, phone number, identity card number or passport number, current location, and self-declaration of infection risks in line with the enforcement of Act 342 [63].

Other similar tracing initiatives implemented by state governments are the Selangkah, PGCare, and Qmunity applications by the Selangor, Penang, and Sarawak state governments, respectively (Table 3). Although they contain similar functions, in-application mechanisms determine the effectiveness of tracing positive cases. A few weeks after the launching of MySejahtera, the two other contract tracing applications developed were also implemented within their respective states. Previously, MySejahtera was not mandatory. However, the Defence Minister officially announced that businesses should utilize MySejahtera as the primary application for contact tracing on 4 August 2020 [64]. The reason underlying this initiative is that the government aspires for a uniform data collection throughout Malaysia because the other applications are limited in terms of coverage and scale. Hence, the government mandated the use of MySejahtera. As MySejahtera is owned and operated by the government, it assures that the collection of the personal information is aligned with the Personal Data Protection Act 2010 (Act 709). Therefore, this application will collect data with the consent of the user and the information collected will not be shared with other parties; thus, it prevents any issues of data misuse [62]. However, citizens are allowed to use other applications for tracing purposes. MySejahtera is widely used across Malaysia and has been key to the detection of spikes in COVID-19 cases. The efforts for close contact tracing are currently going well because 23 million Malaysians and 1 million business premises are using MySejahtera, which has successfully detected 9167 positive cases [65]. Along with the promotion of the application, the government has provided a RM-50 (USD 12) incentive under the National Economy Recovery Plan [66].

As the pandemic persists in the VUCA state, advances in the big data approach will be beneficial for policymakers in formulating sound science- and data-driven policies. In an evolutionary governance, the government is mainly backed by science-based decision making. As the big data approach engages a broad scale of participants, it offers a better understanding for policymakers in demonstrating better leadership and efficiency in formulating policies. This notion is possible when all of the technologies are integrated into a single platform of networks to produce a large pool of database that can offer policymakers with more options [67]. The involvement of experts in synthesizing data will create a new discourse among institutions, which will later balance the power distribution between experts and policymakers in the process of policymaking [39,40].

**Table 3:** The comparison of COVID-19 contact tracing applications in Malaysia.

Items	MySejahtera 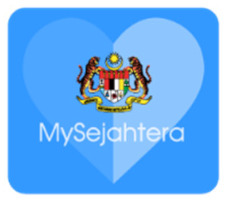	Selangkah 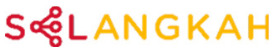	PGCare 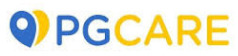	Qmunity 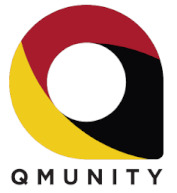
Organizer	Federal government	State government (Selangor)	State government (Penang)	State government (Sarawak)
Date of launched	20 Apr–now	5 May–now	15 May–31 Aug 2020	9 Apr–now
Status	Active	Active	Deactivated	Active
Coverage	Whole Malaysia	Restricted to Selangor	Restricted to Penang	Restricted to Sarawak
Registration document	Identity card number/Passport number Phone number	Phone number	Phone number	Identity card number/Passport number Phone number
Function	Contact tracing	Contact tracing	Contact tracing	Contact tracing
Type of data collected	Name Date of visit Time of visit The place that was checked into Phone number Risk and symptom information	Name Date of visit Time of visit The place that was checked into Phone number	Name Date of visit Time of visit The place that was checked into Phone number Risk and symptom information	Name Identity card number/Passport number Date of visit Time of visit The place that was checked into Phone number Body temperature
Language	Malay English	Malay English Mandarin	Malay English	English Malay
Security	Phone number verification	Not Available	Phone number verification	Phone number verification Face recognition with and without a mask
Additional features	Information on nearby clinics Hotspot areas nearby Latest info of current COVID-19 condition General statistics on COVID-19 cases	Show the average crowd trends of the premises	Not Available	Latest info of current COVID-19 condition General statistics on COVID-19 cases Emergency contacts
Check-in history	Yes	No	No	Yes

Sources: MySejahtera [62], Selangkah [68], PGCare [69], Qmunity [70].

### 3.3. Risk-Based Measures

Nine months after the implementation of the MCO, the number of positive COVID-19 cases in Malaysia continues to fluctuate. The third wave of local infections is ongoing. Hence, the government should ensure that the mitigation measures are flexible and adaptive to the risks of infection. One of the recommended measures in handling the VUCA state is the use of the risk-based approach, which focuses on activities or events that may lead to harmful consequences, whose severity is mainly dependent on the stimulus acting upon it [46].

**Figure 3:**
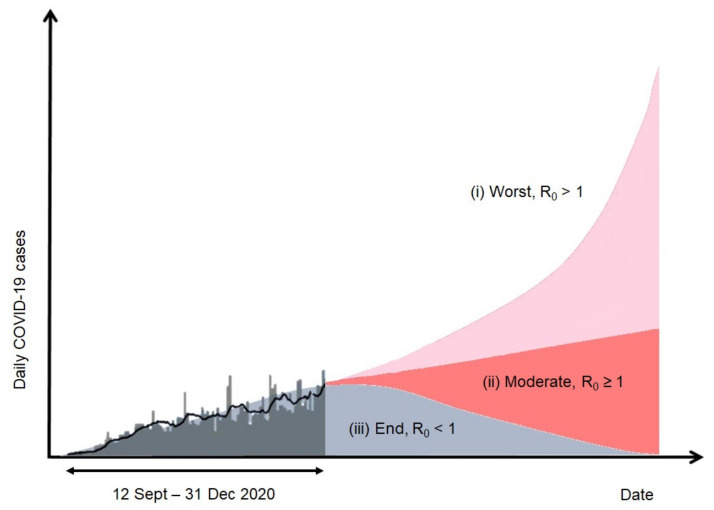
The SEIR model of COVID-19 forecast cases in Malaysia (12 September–31 December 2020) [13].

In the context of the pandemic, risk is defined as the possibility that activities in the community lead to intractable COVID-19 infection. The risk-based approach relies on a numerical assessment of the potential threats of COVID-19 infection during human activities. Figure 3 demonstrates the susceptible–exposed–infectious–removed (SEIR) model reported by the MOH Malaysia in predicting trends in COVID-19 [13]. The model aims to measure the number of infected, recovered, and deceased individuals based on number of contacts, probability of disease transmission, incubation time, recovery rate, and fatality rate [71]. The intensity of the outbreak is assessed based on the magnitude of the *R*_0_ value, which predicts the average number of secondary cases of infection from a positive case using the SEIR model [71,72]. The model posits that an outbreak is expected to continue if *R*_0_ > 1 either being moderate or worst, and end if *R*_0_ < 1 [73]. However, the observed trend is still under control. The *R*_0_ value is influenced by population density (e.g., rural vs. urban) and social structure (e.g., integrated vs. segregated) [74]. Hence, to operationalize risk-based measures, iterative and flexible adaptation plans should be crafted based on the SEIR model to establish SOPs as preventive measures. For example, the increase of COVID-19 cases in the second wave that started on 12 September 2020 resulted in a slight increase of the *R*_0_ value; if the number of COVID-19 cases keeps increasing, the *R*_0_ value increases as well. This indicates that the pandemic situation is marching toward moderate or worst paths against which the government should take proactive and prompt actions to flatten the curve.

Nevertheless, the current policies, strategies, plans, procedures, and practices are mainly reliant on past experiences. The established SOP should not be viewed as a panacea because it may be unable to cope with the pace of unexpected events and changes during the pandemic. Moreover, the risks of COVID-19 vary sociogeographically. Thus, mechanisms for managing risks should be entirely dependent on the respective localities. To ensure the effectiveness of the SOP, it should be flexible and adaptive because it is completely based on the risks of the area. However, errors produced in implementing the SOP will reduce its effectiveness in mitigating the virus. Therefore, evolutionary governance is extremely helpful in understanding the ramifications of laws, rules, and plans as formal bodies. To this end, current policies or assessments, existing scientific papers, and local portrayals should not be considered fully reliable because a standard formula or instrument is insufficient for strengthening the current SOP or addressing social problems [39,40]. Hence, the need to reevaluate the credibility of the use of SOPs as a means of mitigating the spread of the pandemic arises.

Another immediate consequence is that policy transfers and emulated best practices are unlikely to function in the expected manner because they are motivated by an understanding of the course of governance in receiving communities. An exception to this notion is that such policies and procedures should be tailored to the needs of specific communities. Therefore, a new cohesive perspective is required such that certain problems and challenges can be easily anticipated in certain governance paths to produce realistic responses. As discussed in Section 3, evolutionary governance should be incorporated into the formulation of policies and procedures to establish an adaptive and evolutionary SOP suitable for the current VUCA trend of the COVID-19 pandemic in Malaysia. With the sound policies and procedures in place, it will allow the communities to play their role in combating the COVID-19 pandemic through their involvement in community-based monitoring initiative as a means of community empowerment.

## 4. Community-Based Monitoring as a Means of Moving Forward in Terms of the Response of the Government to the Pandemic and the New Normal

To end the RMCO in Malaysia, the final criterion that should be met is community empowerment [75]. In other words, leaders should play a role in empowering communities by taking responsibility to protect themselves from infection. The main agenda is to increase the awareness of the community and educate them to be more responsible and adhere to the preventive measures. Malaysia is a multiethnic and multicultural country. Its citizens are deemed to be collectivistic and relationship-oriented individuals who belong to and are embedded within certain groups, such as family, village, or social groups [76]. These close relationships create mutual and reciprocal obligation between group members and have been influential in uniting communities, especially through difficult times. Moreover, the interpersonal influence of community leaders is extremely strong and plays a significant role in establishing social relationships among group members [77].

Amid the pandemic, communities are as equally important as the frontliners who battle the virus. Communities are crucial in determining the effectiveness of the government’s initiatives [78]. Therefore, previous studies suggested that governance should not be viewed as an orderly series of top-down decision-making mechanisms but as an act of leadership in a scarcely manageable emergence mechanism, where components and systems engage in synchronic or diachronic relationships [39,40]. Community leaders may focus their effort in communicating the risks of infection, strategies to avoid risks, and, importantly, adherence to the new norm of living. Although the focus of such efforts is small-scale and at the grassroots level, it will gradually become significant when members of the communities play their role in helping the country combat COVID-19. Therefore, incorporating the communities into the management of the outbreak is not only a democratizing act but also a promotion of values among the communities. Community-based monitoring as a tool for facilitating the abovementioned objectives may aid the government in empowering communities toward an independent and responsible society.

### 4.1. Approach

In general, the principal understanding of public participation in decision making is related to the right of people to be heard as a part of the democratic process [79]. Overtime, numerous initiatives existed on generating the commitment of communities to the process of collective decision making, which emerged as a tool for community empowerment. This process includes community-based monitoring, citizen science, community science, and other initiatives that share the same vision. The differences between these initiatives can be determined through a continuum level of the communities’ commitment to the program [80,81]. However, the paper does not aim to discuss the differences between these initiatives. Rather, focus is given to community-based monitoring and how such an effort can be integrated into the current pandemic as a response to governance in the new normal.

Community-based monitoring is a social practice where communities collaborate to monitor, track, and respond to specific concerns, especially environmental monitoring [82]. However, the literature provides a wide range of connotations regarding the definition of community-based monitoring, which may be interchangeably used with other terms for similar procedures [83]. In other words, community-based monitoring should be viewed as a method for encouraging local communities to address local issues by cooperating with researchers from diverse disciplines and working entirely in monitoring systems for the well-being of the community. Additionally, this initiative has been practiced for monitoring healthcare issues among communities, especially those in developing countries [84,85]. This practice has been proved to improve community health, especially the delivery of health services in rural communities [86].

The basic principle of community-based monitoring is the engagement of the community in the process of monitoring, which includes regular meetings between researchers and communities, face-to-face meetings and the hands-on approach [87,88,89]. However, in the context of COVID-19 pandemic, people are advised to stay at home and practice social distancing, which renders community-based monitoring difficult. At this point, the approach for community-based monitoring activities should be adaptive to the current situation. In social distancing, for example, all social activities were halted globally. Therefore, using technology for community-based monitoring is a viable option for communities because mobile phones and the Internet are widely used. Moreover, the availability of existing social media platforms and the vast development of mobile applications may realize this initiative. In this manner, participation from communities is increased as all members are eligible to join and free to select their respective roles in monitoring the neighborhood during the pandemic.

The study takes MySejahtera application as an example, as it is widely used within Malaysia and the government mandates the use of MySejahtera. The application can be considered a community-based monitoring initiative. The features developed by the government for contact tracing are commendable. However, the application restricts and limits interactions between citizens and the government. In this regard, the government emphasizes that empowering communities denotes two-way communication. In contrast, the application observes one-way communication, such that citizens contribute to the community by merely using the application. Thus, the outcome of data collected and analyzed and the impact of such data are no longer of interest to them. For this reason, a dynamic and close movement tracing is required. However, MySejahtera uses a static monitoring mode, which indicates that the data are used to chase after the virus instead of moving ahead and containing it before it spreads. Therefore, improvements in the features of the application are required to fully utilize the current government initiative in a manner that will reflect the effective criteria for community-based monitoring.

### 4.2. Criteria

An integrated tracing methodology and mechanism is required to step ahead of the virus spread and eventually end the outbreak. In the subsequent sections, this article provides a few recommendations to enhance the communities’ role through community-based monitoring using MySejahtera, based on the four criteria for community-based monitoring proposed in [90] that will improve the effectiveness of the application.

#### 4.2.1. Efficacy

Efficacy in community-based monitoring heavily relies on the implementation mechanism of monitoring. The main focus of community-based monitoring is participation among communities to strategize the entire process of monitoring. Creating two-way communication between citizens and the government can be achieved by steering the power to citizens. As such, MySejahtera should incorporate a new interactive feature that enables users to report issues related to COVID-19 monitoring. The study suggests four roles that communities can play, namely (i) leaders, (ii) doers, (iii) investigators, or (iv) reporters. Each role is significant, and the only difference is the level of participation. Typically, the highest level of participation is expected from a community leader, whereas a reporter represents the least participation. For instance, if an individual in the community breached the SOP, then community members may lodge a report through the application to alert the authority to take action.

Moreover, providing detailed information about a COVID-19 hotspot within the radius of the respective communities is essential. Information should include the reported number of positive cases and number of individuals undergoing quarantine within an area. With the given information, governments, businesses, and NGOs will gain the capacity to provide targeted financial and psychosocial support to communities at risk, such as periodical checkups, ensuring the awareness of communities regarding the current situation or other related information and providing aid for vital items if necessary. For instance, communities may act as a support system to the affected members or families undergoing home quarantine by helping them stay motivated and undergo a smooth quarantine without violating the SOP. Moreover, this strategy can ease the workload on health care teams in monitoring affected persons.

#### 4.2.2. Technicality

The pandemic continues to evolve and may present further unprecedented challenges, the tracing application should be regularly updated in line with the current situation of the pandemic. Certain features of MySejahtera should be updated on a regular basis, such as the questionnaire on self-check risks and information on COVID-19 hotspots. The reason behind this logic is that the questions on the self-check risk status are general and concentrated only on the risks of COVID-19 infection. However, medical studies on the effects of COVID-19 on patients with hypertension and diabetes found that these patients are at a significantly high risk of mortality if COVID-19 is contracted [91,92]. Hence, the questions for the self-check risk status should include health background, such that individuals with chronic diseases and vulnerable immune systems can obtain a precise risk assessment.

In addition, the method used in MySejahtera to determine individual risk is dependent on the age of users. However, the health risk of an individual cannot be generalized by considering only the age. De Venecia et al. [93] demonstrated an increase in younger adults developing the risk of hypertension, including those with obesity, diabetes mellitus, and renal diseases. This finding supports the fact that a detailed health background is essential for determining the risk status of an individual. Conversely, detailed information may increase the validity of data collected. In other words, if an individual contracts COVID-19, then such information will alert health care teams to prioritize at-risk patient through appropriate medical intervention, thus saving lives.

Furthermore, MySejahtera operates in the static monitoring mode and only collects the check-in location of users to monitor movement. Therefore, the application will trace individuals on the list of those who were at the same location visited by an individual with COVID-19. However, certain places do not enable MySejahtera check-in, which renders contact tracing difficult and thus affects the effectiveness of the application. As such, the application should feature an active monitoring mechanism to speed the process of contact tracing up. In this regard, MyTrace is an active monitoring application developed by the government under the Ministry of Science and Innovation (MOSTI), which was embedded in MySejahtera. However, the user should install both applications separately, which requires the simultaneous operation of the two applications. MyTrace acts as a preventive countermeasure and contact tracing for COVID-19 using low-energy Bluetooth technology. The participating devices exchange proximity information whenever one device detects another nearby device installed with MyTrace [94]. Active monitoring using Bluetooth technology enables the application to consistently run. Although MyTrace was developed by the government, however, its use is voluntary, which renders it less effective because installation is also voluntary. Therefore, to enable synergy between the two applications, the study suggests the integration of MyTrace features into MySejahtera to increase application usability as a means of avoiding potential transmission, especially from patients with asymptomatic COVID-19.

#### 4.2.3. Feedback

Constant engagement with communities will foster trust, which can be achieved through reporting and review. In the context of MySejahtera, one of the feedback mechanisms that can be improved is the constant notification of possible risks to the communities. For instance, the application could notify users if they are approaching or entering red zones or COVID-19 hotspots to enable them to stay alert in their surroundings and avoid any form of transmission. Another possible feature that can be added into MySejahtera is notifications about the SOP when users check into certain premises, as the reports point to the increasing rates of noncompliance with the SOP [95]. The SOP displayed should depend on the type of premises or places visited. For instance, if users were to enter a restaurant and check into the location through MySejahtera, the users should instantly receive a notification from the application informing about the current SOPs observed at the restaurant. Users are then required to check on a box indicating observance of the SOP and thus avoid being charged for violation of the SOP.

#### 4.2.4. Sustainability

Reviewing the current trend of COVID-19 infection worldwide, no party can determine the duration of the pandemic. Future events and beyond will largely depend on the production and effectiveness of vaccines and the efficiency of the governments in implementing preventive strategies to handle the pandemic. In the context of the sustainability of community-based monitoring via MySejahtera, the study proposes the categorization of the COVID-19 pandemic into three scenarios, namely worst case (*R*_0_ > 1), status quo (*R*_0_ ≥ 1), and best case (*R*_0_ < 1).

Under the worst-case scenario, COVID-19 cases will continue to increase rapidly in the absence of effective vaccines or lasting immunity. This tendency will lead to an extensive circulation of the virus, which can cause the pandemic to persist [96]. The same is true for the status quo, where the number of cases continues to increase on a daily basis. The only difference between them is the frequency of newly reported cases. In the worst-case scenario, the trend pertains to a skyrocketing number of daily reported cases, whereas the status quo indicates a slight increase in daily reported cases. For both cases, the contagion rate in the communities is high and infections are most likely to spread rapidly within a short time. Thus, the current passive monitoring of MySejahtera for contact tracing will be rendered ineffective and slow. As suggested in the previous section, active surveillance should be included as an additional feature or set of features for data collected from contact tracing. When communities are directly involved in active surveillance, rapid containment of the infection and reduction of risks is possible. In this manner, the government plays a major role in coordinating various stakeholders of formal and informal institutions to adapt to the proposed changes. A continuous transformation of knowledge configuration between actors can lead to success in managing the current disaster.

Under the best-case scenario, the pandemic will end with or without vaccines being applied. However, will the tracing application remain relevant? Should MySejahtera be rescinded once the pandemic is over? Currently, the MySejahtera application is widely used within the country; the government could use this application as a vaccination passport, thus increasing the public dependability on this application. If the pandemic comes to an end, the government should utilize this opportunity by reconfiguring the application for other public health monitoring purposes—for instance, dengue. Falling under the same category of communicable diseases as COVID-19, dengue continues to record thousands of infected cases and mortalities per year in Malaysia. In 2020, a total of 50,511 infections and 88 fatalities from dengue cases were reported on 1 Jan and 13 June, respectively [97]. The tracing mechanism in the application will be beneficial by increasing the communities’ awareness about dengue hotspots. Accordingly, appropriate locality-based prevention strategies can be implemented in these areas. Hence, the government should consider reusing and improvising MySejahtera for community-based monitoring, especially for the sustainability of public health.

## 5. Conclusions

The COVID-19 pandemic has disrupted the public health systems in many countries due to its high contagion rate and tendency to be transmitted from individuals with and without symptoms. As such, determining the risks of infection within communities has been difficult. Malaysia has implemented phases of the MCO to mitigate the spread of COVID-19 within communities. However, such preventive measures via partial lockdown exerted a tremendous impact on the socioeconomic aspect. To battle the pandemic, an evolutionary governance should underpin mitigation strategies to address the current volatile, uncertain, complex, and ambiguous situation. Moreover, scientific research on COVID-19 based on facts and factual data is essential for the formulation of science-driven decisions of the government.

Policies derived from science-driven decision making requires high-quality and reliable data for the analysis and interpretation of experts in formulating risk-based mitigation measures. Subsequently, to enable such measures to be operational, iterative and flexible adaptation plans should be crafted based on the data and model for establishing the SOP. In Malaysia, the mobile application MySejahtera has been introduced to empower communities in collecting data as a form of community-based monitoring with the support of industries and businesses within the premises of such communities. However, to ensure the effectiveness of this tool for community-based monitoring, we recommend collaboration between researchers and communities to improve the method for data collection.

In this study, we highlighted the four criteria of community-based monitoring, namely efficacy, technicality, feedback, and sustainability as means of improving the bottom-up data feed-in through the government and the assistance of NGOs to formulate sound decisions and policies for battling the COVID-19 pandemic. In summary, community-based monitoring is expected to be instrumental as a strategy for bridging communities with the government not only through data collection but also through participation in governance for the well-being of the country.

## Figures and Tables

**Table 2:** The different approaches taken by the Malaysian government in containing the local infection of COVID-19.

	EMCO	TEMCO	AEMCO
Coverage area	Specific localities, such as district or a village	Small and specific areas, such as residential complex	Specific localities, such as district or a village
Days of quarantine	14 days	28 days	14 days
Standard Operating Procedure (SOP)	All businesses ordered to close All education institutions ordered to close All religious places ordered to close Food supplies and essential items provided by the government Working residents not allowed to leave the area Permission to work only for essential services with the approval of the authorities	All businesses ordered to close All education institutions ordered to close All religious places ordered to close Food supplies and essential items provided by the government Working residents not allowed to leave the area Permission to work only for essential services with the approval of the authorities	Essential businesses allowed to operate from 8 a.m.–8 p.m. All education institutions ordered to close All religious places ordered to close Only drive-thru, takeaway, or delivery of food allowed Only one person per family allowed to get the food supplies The daily market allowed to operate between 6 a.m.–2 p.m. Working residents not allowed to leave the area Permission to work only for essential services with the approval of the authorities
Enforcement	Residents strictly needed to stay at home	Residents strictly needed to stay at home	Residents allowed to move within the area
Screening and testing for COVID-19	Home to the home screening	Home to the home screening	All residents need to undergo screening at the nearby clinics

EMCO—Enhanced Movement Control Order; TEMCO—Targeted Enhanced Movement Control Order; AEMCO—Administrative Enhanced Movement Control Order. Source: [18].

## Data Availability

Data were derived from open and public resources and made available with this article. The number of COVID-19 reported cases in Malaysia are retrievable in the Ministry of Health Malaysia’s website, https://kpkesihatan.com/ (accessed on 31 December 2020).
